# Numerical Investigation of the Effect of Unsteadiness on Three-Dimensional Flow of an Oldroyb-B Fluid

**DOI:** 10.1371/journal.pone.0133507

**Published:** 2015-07-21

**Authors:** S. S. Motsa, Z. G. Makukula, S. Shateyi

**Affiliations:** 1 School of Mathematics, Statistics and Computer Science, University of KwaZulu-Natal, Private Bag X01, Scottsville 3209, Pietermaritzburg, South Africa; 2 Department of Mathematics & Applied Mathematics, University of Venda, Private Bag X5050, Thohoyandou 0950, South Africa; China University of Mining and Technology, CHINA

## Abstract

A spectral relaxation method used with bivariate Lagrange interpolation is used to find numerical solutions for the unsteady three-dimensional flow problem of an Oldroyd-B fluid with variable thermal conductivity and heat generation. The problem is governed by a set of three highly coupled nonlinear partial differential equations. The method, originally used for solutions of systems of ordinary differential equations is extended to solutions of systems of nonlinear partial differential equations. The modified approach involves seeking solutions that are expressed as bivariate Lagrange interpolating polynomials and applying pseudo-spectral collocation in both independent variables of the governing PDEs. Numerical simulations were carried out to generate results for some of the important flow properties such as the local skin friction and the heat transfer rate. Numerical analysis of the error and convergence properties of the method are also discussed. One of the benefits of the proposed method is that it is computationally fast and gives very accurate results after only a few iterations using very few grid points in the numerical discretization process.

## Introduction

Oldroyd-B fluids are non-Newtonian viscoelastic fluids classified as the rate type model. Models of the rate type are suitable for describing many of the non-Newtonian characteristics shown by polymeric materials such as stress-relaxation, normal stress differences in simple shear flows and creep [1, 2]. The Oldroyd-B model is mostly applicable in modeling the response of dilute polymeric solutions and has since received significant attention of rheologists. However, rate type models can not capture the complex rheological behaviour of many real fluids, such as blood in which the non-Newtonian viscosity effects are of major importance [2]. Non-Newtonian fluids find applications in industry and technology, such as bio-medicine, chemical engineering, food stuff, pharmaceutical industries, production of plastic sheet, extrusion of polymers through a slit die in polymer industry and many others [3–5].

Hayat et al. [6] studied the flow of an electrically conducting, incompressible and Oldroyd-B fluid between two infinitely extended non-conducting parallel plates in presence of a uniform transverse magnetic field fixed relative to the fluid. Analytical solutions of the flow models were obtained for both the steady and unsteady case. Fetecau et al. [7] presented an analytical solution for the velocity field and the adequate shear stress corresponding to the decay of a potential vortex in a generalized Oldroyd-B fluid by means of Hankel and Laplace transforms. The Hankel and Laplace transforms were also used by Rubbab et al. [1] and Tong et al. [8] to find analytical solutions for the unsteady flow of an incompressible generalized Oldroyd-B fluid in an infinite circular cylinder and unsteady helical flows of a generalized Oldroyd-B fluid with fractional calculus. The Weber transforms were used in addition to the Hankel and Laplace transforms by Qi and Jin [9] for solutions of the unsteady helical flows of a generalized Oldroyd-B fluid between two infinite coaxial cylinders and within an infinite cylinder.

The homotopy analysis method (HAM) was used by Hayat et al. [10] to study the thermal radiation effects on the two-dimensional magnetohydrodynamic (MHD) flow of an Oldroyd-B fluid in the presence of Joule heating and thermophoresis. Using finite element methods, Pires and Sequeira [2] investigated the behavior of fully developed flows of shear-thinning generalized Oldroyd-B fluids in curved pipes with circular cross-section and arbitrary curvature ratio, for a prescribed pressure gradient. Liu et al. [11] used Laplace transforms for an analytical study for the magnetohydrodynamic (MHD) flow of a generalized Oldroyd-B fluid. Hayat et al. [3] found series solutions using the HAM for a three-dimensional flow of an Oldroyd-B fluid over a stretching surface in the presence of convective boundary conditions. A numerical study was carried out by Nadeem et al. [12] using the fourth-fifth order Runge-Kutta-Fehlberg method, to study the Oldroyd-B nanofluid flow model over a stretching sheet. Recently, Sajid et al. [13] used finite difference method to carry out a numerical study for a mixed convection in the stagnation-point flow of an Oldroyd-B fluid over a stretching sheet. Khan et al. [5] used the HAM to study the free convective boundary-layer flow of three-dimensional Oldroyd-B nanofluid flow over a stretching sheet. Hayat et al. [4] also used the HAM to investigate the three-dimensional flow of non-Newtonian fluid over a bidirectional stretching surface with heat transfer.

In the above studies and more, not cited in this article, little attention has been given to unsteady flows of the Oldroyd-B fluid, especially three-dimensional flows. In this study we carry out a numerical investigation of the unsteady three-dimensional flow of an Oldroyd-B fluid with variable thermal conductivity and heat generation/absorption. The work is an extension of the steady state flow considered by Shehzad et al. [14]. The Homotopy analysis method was implemented in their study to develop series solutions to the governing flow and energy equations. In this work we use the bivariate spectral relaxation method for the first time in higher order systems of nonlinear partial differential equations (PDEs). The method combines ideas of the Gauss-Seidel approach to decouple the nonlinear system of PDEs into a sequence of linear partial differential equations which are then solved using the Chebyshev spectral collocation method with bivariate Lagrange interpolation polynomials as basis functions. Bivariate Lagrange interpolation has been used successfully in a recent study by Motsa et al. [15]. The polynomials were used in a quasilinearisation scheme to approximate solutions of nonlinear evolution parabolic PDEs. Comparison with their exact solutions proved that the method gives accurate solutions in a computationally efficient manner. In this work the bivariate Lagrange spectral collocation approach is used with relaxation on a coupled system of PDEs.

## Governing equations

Consider the unsteady three-dimensional flow of an incompressible Oldroyd-B fluid. The flow is caused by a stretched surface at *z* = 0 and occupies the *z* > 0 domain. Thermal conductivity is assumed to be a linear function of temperature and the ambient fluid temperature is represented by *T*_∞_. Effects of heat generation or absorption on the boundary layer flow are taken into consideration. Following [14], the governing equations for unsteady three-dimensional flow and heat transfer are given as

$$\frac{\partial u}{\partial x}+\frac{\partial v}{\partial y}+\frac{\partial w}{\partial z}=0,$$ (1)

$$\begin{array}{l} \frac{\partial u}{\partial t}+u\frac{\partial u}{\partial x}+v\frac{\partial u}{\partial y}+w\frac{\partial u}{\partial z}+\lambda_{1}(u^{2}\frac{\partial^{2}u}{\partial x^{2}}+v^{2}\frac{\partial^{2}u}{\partial y^{2}}+w^{2}\frac{\partial^{2}u}{\partial z^{2}}+2uv\frac{\partial^{2}u}{\partial x\partial y}+2vw\frac{\partial^{2}u}{\partial y\partial z}\\ +2uw\frac{\partial^{2}u}{\partial x\partial z})\\ =\nu \left\{\frac{\partial^{2}u}{\partial z^{2}}+\lambda_{2}\left(u\frac{\partial^{3}u}{\partial x\partial z^{2}}+v\frac{\partial^{3}u}{\partial y\partial z^{2}}+w\frac{\partial^{3}u}{\partial z^{3}}-\frac{\partial u\partial^{2}u}{\partial x\partial z^{2}}-\frac{\partial u\partial^{2}v}{\partial y\partial z^{2}}-\frac{\partial u\partial^{2}w}{\partial z\partial z^{2}}\right)\right\}, \end{array}$$ (2)

$$\begin{array}{l} \frac{\partial v}{\partial t}+u\frac{\partial v}{\partial x}+v\frac{\partial v}{\partial y}+w\frac{\partial v}{\partial z}+\lambda_{1}(u^{2}\frac{\partial^{2}v}{\partial x^{2}}+v^{2}\frac{\partial^{2}v}{\partial y^{2}}+w^{2}\frac{\partial^{2}v}{\partial z^{2}}+2uv\frac{\partial^{2}v}{\partial x\partial y}+2vw\frac{\partial^{2}v}{\partial y\partial z}\\ +2uw\frac{\partial^{2}v}{\partial x\partial z})\\ =\nu \left\{\frac{\partial^{2}v}{\partial z^{2}}+\lambda_{2}\left(u\frac{\partial^{3}v}{\partial x\partial z^{2}}+v\frac{\partial^{3}v}{\partial y\partial z^{2}}+w\frac{\partial^{3}v}{\partial z^{3}}-\frac{\partial v\partial^{2}v}{\partial x\partial z^{2}}-\frac{\partial v\partial^{2}v}{\partial y\partial z^{2}}-\frac{\partial v\partial^{2}w}{\partial z\partial z^{2}}\right)\right\}, \end{array}$$ (3)

$$\frac{\partial T}{\partial t}+\rho C_{p}(u\frac{\partial T}{\partial x}+v\frac{\partial T}{\partial y}+w\frac{\partial T}{\partial z})=\frac{\partial }{\partial z}(\kappa \frac{\partial T}{\partial z})+Q\left(T-T_{\infty }\right).$$ (4)

We note here that without time dependence, equation system Eqs (1)–(4) reduces to the equations considered by Shehzad et al. [14]. In Eqs (1)–(4), *u*, *v* and *w* are the velocity components in the *x*−, *y*− and *z*− directions respectively. The relaxation time is represented by *λ*_1_ while the retardation time by *λ*_2_. The fluid temperature is denoted by *T*, *t* is the time, *k* is the thermal conductivity of fluid, *σ* denotes thermal diffusivity of the fluid, *ν* (*μ*/*ρ*) the kinematic viscosity, *μ* the dynamic viscosity of the fluid, *ρ* the density of fluid and *Q* the heat generation/absorption parameter. The fluid flow is subject to following initial and boundary conditions

$$\begin{array}{ccc} & t<0: & \;u=v=w=0,\;T=T_{\infty },\;\text{for}\;\text{any}\;x,y,z,\\ & t\geq 0: & \;u=ax,\;v=by,\;w=0,\;T=T_{w},\;\text{at}\;z=0,\\ & u\to 0, & \;v\to 0,T\to 0,\;\text{as}\;z\to \infty . \end{array}$$ (5)

where *a* and *b* are the velocity gradients in the *x*− and *y*− directions respectively. Following [14, 16] we introduce the following similarity transformations in Eqs (2)–(4)

$$\begin{array}{ccc} & \xi =1-e^{-\tau }, & \;\tau =a\;t,\;\eta ={\left(\frac{a}{\nu }\right)}^{\frac{1}{2}}\xi^{-\frac{1}{2}}z,\;u=axf^{\prime }\left(\xi ,\eta \right),\\ & v=ayg^{\prime }\left(\xi ,\eta \right), & \;w=-{\left(a\nu \right)}^{\frac{1}{2}}\xi^{\frac{1}{2}}\left(f\left(\xi ,\eta \right)+g\left(\xi ,\eta \right)\right),T=T_{\infty }+\left(T_{w}-T_{\infty }\right)\theta \left(\xi ,\eta \right), \end{array}$$ (6)

resulting into the system of nonlinear PDEs given by

$$\begin{array}{cc} & f^{\prime \prime \prime }+\frac{\eta }{2}\left(1-\xi \right)f^{\prime \prime }+\xi [\left(f+g\right)f^{\prime \prime }-{f^{\prime }}^{2}+\beta_{1}\left(2\left(f+g\right)f^{\prime }f^{\prime \prime }-{\left(f+g\right)}^{2}f^{\prime \prime \prime }\right)]\\ & +\beta_{2}\left(\left(f^{\prime \prime }+g^{\prime \prime }\right)f^{\prime \prime }-\left(f+g\right)f^{iv}\right)=\xi \left(1-\xi \right)\frac{\partial f^{\prime }}{\partial \xi }, \end{array}$$ (7)

$$\begin{array}{cc} & g^{\prime \prime \prime }+\frac{\eta }{2}\left(1-\xi \right)g^{\prime \prime }+\xi [\left(f+g\right)g^{\prime \prime }-{g^{\prime }}^{2}+\beta_{1}\left(2\left(f+g\right)g^{\prime }g^{\prime \prime }-{\left(f+g\right)}^{2}g^{\prime \prime \prime }\right)]+\\ & \beta_{2}\left(\left(f^{\prime \prime }+g^{\prime \prime }\right)g^{\prime \prime }-\left(f+g\right)g^{iv}\right)=\xi \left(1-\xi \right)\frac{\partial g^{\prime }}{\partial \xi }, \end{array}$$ (8)

$$\left(1+\varepsilon \theta \right)\theta^{\prime \prime }+Pr\frac{\eta }{2}\left(1-\xi \right)\theta^{\prime }+\xi Pr\left(f+g\right)\theta^{\prime }+\varepsilon \theta^{\prime 2}+\xi PrS\theta =Pr\xi \left(1-\xi \right)\frac{\partial \theta }{\partial \xi },$$ (9)

with the corresponding boundary conditions

$$f\left(\xi ,0\right)=0,\;f^{\prime }\left(\xi ,0\right)=1,\;f^{\prime }\left(\xi ,\infty \right)=0,$$ (10)

$$g\left(\xi ,0\right)=0,\;g^{\prime }\left(\xi ,0\right)=\beta ,\;g^{\prime }\left(\xi ,\infty \right)=0,$$ (11)

θ(ξ,0)=1,θ(ξ,∞)=0. (12)

We remark that, Eqs (7)–(9) reduce to the steady state flow equations when *ξ* = 1 as reported in [14]. In Eqs (7)–(9), *β*_1_ and *β*_2_ are Deborah numbers, *β* is a ratio of stretching rates parameter, *Pr* is the Prandtl number, and *S* the heat generation/absorption parameter given by

$$\beta_{1}=\lambda_{1}a,\;\beta_{2}=\lambda_{1}a,\;\beta =\frac{b}{a},\;Pr=\frac{\rho C_{p}\nu }{\kappa },\;S=\frac{Q}{\rho aC_{p}}.$$ (13)

The important physical parameters for the type of boundary layer flow under consideration are the skin-friction coefficient, Nusselt number and Sherwood number. From the velocity field, the shearing stress at the surface can be obtained, which in the non-dimensional form (skin-friction coefficient) in the *x* and *y* directions using Eq (7) is given by

$$C_{f}^{x}={\left(\xi Re_{x}\right)}^{-\frac{1}{2}}f^{\prime \prime }\left(\xi ,0\right),\;C_{f}^{y}={\left(\xi Re_{x}\right)}^{-\frac{1}{2}}g^{\prime \prime }\left(\xi ,0\right),$$ (14)

where *Re*_*x*_ = *ax* ^2^/*ν* is the local Reynolds number.

Using the temperature field, the heat transfer coefficient at the surface can be obtained, which in the non-dimensional form, in terms of the Nusselt number, is given by

$$Nu_{r}=Nu_{x}{\left(\xi Re_{x}\right)}^{-\frac{1}{2}}=-\theta^{\prime }\left(\xi ,0\right).$$ (15)

## The bivariate spectral relaxation method (BSRM)

In this section, we describe the application of the bivariate spectral relaxation method (BSRM) to find numerical solutions of the system of governing Eqs (7)–(9). The method applies the Gauss-Siedel relaxation idea to rearrange and decouple the system to form a linear sequence of partial differential equations that are solved in succession over a number of iterations. Consequently, re-arranging Eqs (7)–(9) and linearising in Gauss-Seidel manner gives

$$a_{1,r}\left(\eta ,\xi \right)f_{r+1}^{\left(iv\right)}+a_{2,r}\left(\eta ,\xi \right)f_{r+1}^{\prime \prime \prime }+a_{3,r}\left(\eta ,\xi \right)f_{r+1}^{\prime \prime }-\xi \left(1-\xi \right)\frac{\partial f_{r+1}^{\prime }}{\partial \xi }=a_{4,r}\left(\eta ,\xi \right),$$ (16)

$$b_{1,r}\left(\eta ,\xi \right)g_{r+1}^{\left(iv\right)}+b_{2,r}\left(\eta ,\xi \right)g_{r+1}^{\prime \prime \prime }+b_{3,r}\left(\eta ,\xi \right)g_{r+1}^{\prime \prime }-\xi \left(1-\xi \right)\frac{\partial g_{r+1}^{\prime }}{\partial \xi }=b_{4,r}\left(\eta ,\xi \right),$$ (17)

$$c_{1,r}\left(\eta ,\xi \right)\theta_{r+1}^{\prime \prime }+c_{2,r}\left(\eta ,\xi \right)\theta_{r+1}^{\prime }+c_{3,r}\left(\eta ,\xi \right)\theta_{r+1}-Pr\xi \left(1-\xi \right)\frac{\partial \theta_{r+1}}{\partial \xi }=c_{4,r}\left(\eta ,\xi \right),$$ (18)

with

$$\begin{array}{ll} & a_{1,r}\left(\eta ,\xi \right)=-\beta_{2}\left(f_{r}+g_{r}\right),\;a_{2,r}\left(\eta ,\xi \right)=1-\beta_{1}\xi {\left(f_{r}+g_{r}\right)}^{2},\\ & a_{3,r}\left(\eta ,\xi \right)=\frac{\eta }{2}\left(1-\xi \right)+\xi \left(f_{r}+g_{r}\right)+2\beta_{1}\xi \left(f_{r}+g_{r}\right)f_{r}^{\prime }+\beta_{2}g_{r}^{\prime \prime },\\ & a_{4,r}\left(\eta ,\xi \right)=\xi f_{r}^{\prime 2}-\beta_{2}f_{r}^{\prime \prime 2},\\ & b_{1,r}\left(\eta ,\xi \right)=-\beta_{2}\left(f_{r+1}+g_{r}\right),\;b_{2,r}\left(\eta ,\xi \right)=1-\beta_{1}\xi {\left(f_{r+1}+g_{r}\right)}^{2},\\ & b_{3,r}\left(\eta ,\xi \right)=\frac{\eta }{2}\left(1-\xi \right)+\xi \left(f_{r+1}+g_{r}\right)+2\beta_{1}\xi \left(f_{r+1}+g_{r}\right)g_{r}^{\prime }+\beta_{2}f_{r}^{\prime \prime },\\ & b_{4,r}\left(\eta ,\xi \right)=\xi g_{r}^{\prime 2}-\beta_{2}g_{r}^{\prime \prime 2},\\ & c_{1,r}\left(\eta ,\xi \right)=1+\varepsilon \theta_{r},\;c_{2,r}\left(\eta ,\xi \right)=Pr[\xi \left(f_{r+1}+g_{r+1}\right)+\frac{\eta }{2}\left(1-\xi \right)],\\ & c_{3,r}\left(\eta ,\xi \right)=PrS\xi ,\;c_{4,r}\left(\eta ,\xi \right)=-\varepsilon \theta_{r}^{\prime 2}. \end{array}$$ (19)

Eqs (16)–(18) form a linear decoupled system of partial differential equations and can be solved iteratively starting from given initial approximations, denoted by *f*_0_, *g*_0_ and *θ*_0_. The iteration is repeated for *r* = 1, 2, …, until approximate solutions that are consistent to within a certain tolerance level are obtained. To solve Eqs (16)–(18) we apply spectral collocation in both the *η* and *ξ* direction with bivariate Lagrange interpolation polynomials as basis functions. Thus, the approximate solution for *g*(*η*, *ξ*), say, takes the form

$$g\left(\eta ,\xi \right)\approx \sum_{m=0}^{N_{x}}\sum_{j=0}^{N_{t}}g\left(\tau_{m},\zeta_{j}\right)L_{m}\left(\tau \right)L_{j}\left(\zeta \right),$$ (20)

which interpolates *g*(*η*, *ξ*) at the collocation points defined by

$$\tau_{i}=\cos(\frac{\pi i}{N_{x}}),\;\;\;\;\zeta_{j}=\cos(\frac{\pi j}{N_{t}}),\;\;\;i=0,1,\ldots ,N_{x};\;\;j=0,1,\ldots ,N_{t}.$$ (21)

The functions *L*_*m*_(*τ*) and *L*_*j*_(*ζ*) are the well-known characteristic Lagrange cardinal polynomials. It is worth remarking that the Chebyshev collocation method requires that the domain of the problem be transformed to [−1, 1] × [−1, 1]. We therefore use simple linear transformation to transform *η* ∈ [0, *η*_∞_] and *ξ* ∈ [0, 1] to *τ* ∈ [−1, 1] and *ζ* ∈ [−1, 1], respectively. Here *η*_∞_ is a finite value that is introduced to facilitate the application of the numerical method at infinity.

Other choices of basis functions can be considered in place of the Lagrange polynomials in Eq (20), but the difference in terms of accuracy of the obtained solutions is minimal. The choice of Lagrange interpolation polynomials given by Eq (20) makes it possible to convert the continuous derivatives to discrete matrix form at the collocation points Eq (21) using the so-called derivative matrices defined in [17, 18]. For example, the derivatives of *g*(*η*, *ξ*) with respect to *η* and *ξ* at the collocation points *τ*_*k*_ and *ζ*_*i*_ are defined as follows:

$${\frac{\partial g}{\partial \eta }|}_{\left(\tau_{k},\zeta_{i}\right)}=\frac{2}{\eta_{\infty }}\sum_{m=0}^{N_{x}}\sum_{j=0}^{N_{t}}g\left(\tau_{m},\zeta_{j}\right)\frac{dL_{m}\left(\tau_{k}\right)}{d\tau }L_{j}\left(\zeta_{i}\right)=DG_{i},$$ (22)

$${\frac{\partial^{2}g}{\partial \eta^{2}}|}_{\left(\tau_{k},\zeta_{i}\right)}=D^{2}G_{i},$$ (23)

$${\frac{\partial g}{\partial \xi }|}_{\left(\tau_{k},\zeta_{i}\right)}=2\sum_{m=0}^{N_{x}}\sum_{j=0}^{N_{t}}g\left(\tau_{m},\zeta_{j}\right)\frac{dL_{j}\left(\zeta_{i}\right)}{d\zeta }L_{m}\left(\tau_{k}\right)=2\sum_{j=0}^{N_{t}}d_{ij}G_{j},$$ (24)

where *d*_*i*,*j*_ (*i*, *j* = 0, 1, …, *N*_*t*_) are entries of the standard Chebyshev differentiation matrix *d* = [*d*_*i*,*j*_] of size (*N*_*t*_ + 1) × (*N*_*t*_ + 1) (see, for example [17, 18]), **D** = (2/*η*_*e*_)[*D*_*r*,*s*_] (*r*, *s* = 0, 1, 2, …, *N*_*x*_) with [*D*_*r*,*s*_] being an (*N*_*x*_ + 1) × (*N*_*x*_ + 1) Chebyshev derivative matrix, and the vector **G**_*i*_ is defined as

$$G_{i}={\left[g_{i}\left(\tau_{0}\right),g_{i}\left(\tau_{1}\right),\ldots ,g_{i}\left(\tau_{N_{x}}\right)\right]}^{T}.$$ (25)

The derivatives of *f* and *θ* can be transformed to discrete matrix form in a similar manner. Applying the spectral collocation method by evaluating Eqs (16)–(18) at the collocation points and making use of the derivative matrices gives,

$$A_{i}F_{r+1,i}-2\xi_{i}\left(1-\xi_{i}\right)\sum_{j=0}^{N_{t}}d_{i,j}F_{r+1,j}=a_{4,r}\left(\xi_{i}\right),$$ (26)

$$B_{i}G_{r+1,i}-2\xi_{i}\left(1-\xi_{i}\right)\sum_{j=0}^{N_{t}}d_{i,j}G_{r+1,j}=b_{4,r}\left(\xi_{i}\right),$$ (27)

$$A_{i}\Theta_{r+1,i}-2Pr\xi_{i}\left(1-\xi_{i}\right)\sum_{j=0}^{N_{t}}d_{i,j}\Theta_{r+1,j}=c_{4,r}\left(\xi_{i}\right),$$ (28)

for *i* = 0, 1, 2, …, *N*_*t*_, where

$$A_{i}=a_{1,r}\left(\xi_{i}\right)D^{4}+a_{2,r}\left(\xi_{i}\right)D^{3}+a_{3,r}\left(\xi_{i}\right)D^{2},$$

$$B_{i}=b_{1,r}\left(\xi_{i}\right)D^{4}+b_{2,r}\left(\xi_{i}\right)D^{3}+b_{3,r}\left(\xi_{i}\right)D^{2},$$

$$C_{i}=c_{1,r}\left(\xi_{i}\right)D^{2}+c_{2,r}\left(\xi_{i}\right)D+c_{3,r}\left(\xi_{i}\right),$$

$$F_{i}={\left[f_{i}\left(\tau_{0}\right),f_{i}\left(\tau_{1}\right),\ldots ,f_{i}\left(\tau_{N_{x}}\right)\right]}^{T},$$

$$\Theta_{i}={\left[\theta_{i}\left(\tau_{0}\right),\theta_{i}\left(\tau_{1}\right),\ldots ,\theta_{i}\left(\tau_{N_{x}}\right)\right]}^{T},$$

$$a_{4,r}\left(\xi_{i}\right)={\left[a_{4,r}\left(\tau_{0},\xi_{i}\right),a_{4,r}\left(\tau_{1},\xi_{i}\right),\ldots ,a_{4,r}\left(\tau_{N_{x}},\xi_{i}\right)\right]}^{T},$$

$$b_{4,r}\left(\xi_{i}\right)={\left[b_{4,r}\left(\tau_{0},\xi_{i}\right),b_{4,r}\left(\tau_{1},\xi_{i}\right),\ldots ,b_{4,r}\left(\tau_{N_{x}},\xi_{i}\right)\right]}^{T},$$

$$c_{4,r}\left(\xi_{i}\right)={\left[c_{4,r}\left(\tau_{0},\xi_{i}\right),c_{4,r}\left(\tau_{1},\xi_{i}\right),\ldots ,c_{4,r}\left(\tau_{N_{x}},\xi_{i}\right)\right]}^{T},$$

and **a**_*m*,*r*_(*ξ*_*i*_), **b**_*m*,*r*_(*ξ*_*i*_), **c**_*m*,*r*_(*ξ*_*i*_) (*m* = 1, 2, 3) are the diagonal matrices of the vectors [*a*_*m*,*r*_(*τ*_0_, *ξ*_*i*_), *a*_*m*, *r*_(*τ*_1_, *ξ*_*i*_), …, *a*_*m*,*r*_(*τ*_*N*_*x*__, *ξ*_*i*_)]^*T*^, [*b*_*m*,*r*_(*τ*_0_, *ξ*_*i*_), *b*_*m*,*r*_(*τ*_1_, *ξ*_*i*_), …, *b*_*m*, *r*_(*τ*_*N*_*x*__, *ξ*_*i*_)]^*T*^ [*c*_*m*,*r*_(*τ*_0_, *ξ*_*i*_), *c*_*m*,*r*_(*τ*_1_, *ξ*_*i*_), …, *c*_*m*,*r*_(*τ*_*N*_*x*__, *ξ*_*i*_)]^*T*^.

After imposing boundary conditions for *i* = 0, 1, …, *N*_*t*_, Eqs (26)–(28) can be written in matrix form as:

$$[\begin{array}{cccc} A_{0,0} & A_{0,1} & \cdots & A_{0,N_{t}}\\ A_{1,0} & A_{1,1} & \cdots & A_{1,N_{t}}\\ \vdots & \vdots & \ddots & \vdots \\ A_{N_{t},0} & A_{N_{t},1} & \cdots & A_{N_{t},N_{t}} \end{array}][\begin{array}{c} F_{r+1,0}\\ F_{r+1,1}\\ \vdots \\ F_{r+1,N_{t}} \end{array}]=[\begin{array}{c} R_{1,0}\\ R_{1,1}\\ \vdots \\ R_{1,N_{t}} \end{array}],$$ (29)

$$[\begin{array}{cccc} B_{0,0} & B_{0,1} & \cdots & B_{0,N_{t}}\\ B_{1,0} & B_{1,1} & \cdots & B_{1,N_{t}}\\ \vdots & \vdots & \ddots & \vdots \\ B_{N_{t},0} & B_{N_{t},1} & \cdots & B_{N_{t},N_{t}} \end{array}][\begin{array}{c} G_{r+1,0}\\ G_{r+1,1}\\ \vdots \\ G_{r+1,N_{t}} \end{array}]=[\begin{array}{c} R_{2,0}\\ R_{2,1}\\ \vdots \\ R_{2,N_{t}} \end{array}],$$ (30)

$$[\begin{array}{cccc} C_{0,0} & C_{0,1} & \cdots & C_{0,N_{t}}\\ C_{1,0} & C_{1,1} & \cdots & C_{1,N_{t}}\\ \vdots & \vdots & \ddots & \vdots \\ C_{N_{t},0} & C_{N_{t},1} & \cdots & C_{N_{t},N_{t}} \end{array}][\begin{array}{c} \Theta_{r+1,0}\\ \Theta_{r+1,1}\\ \vdots \\ \Theta_{r+1,N_{t}} \end{array}]=[\begin{array}{c} R_{3,0}\\ R_{3,1}\\ \vdots \\ R_{3,N_{t}} \end{array}],$$ (31)

where

$$A_{i,i}=A_{i}-2\xi_{i}\left(1-\xi_{i}\right)d_{i,i}D,\;\;\;i=0,1,\ldots ,N_{t}-1,$$ (32)

$$B_{i,i}=B_{i}-2\xi_{i}\left(1-\xi_{i}\right)d_{i,i}I,\;\;\;i=0,1,\ldots ,N_{t}-1,$$ (33)

$$C_{i,i}=C_{i}-2Pr\xi_{i}\left(1-\xi_{i}\right)d_{i,i}I,\;\;\;i=0,1,\ldots ,N_{t}-1,$$ (34)

$$A_{i,j}=-2\xi_{i}\left(1-\xi_{i}\right)d_{i,j}I,\;\;\;\text{when}\;i\neq j,$$ (35)

$$B_{i,j}=-2\xi_{i}\left(1-\xi_{i}\right)d_{i,j}I,\;\;\;\text{when}\;i\neq j,$$ (36)

$$C_{i,j}=-2Pr\xi_{i}\left(1-\xi_{i}\right)d_{i,j}I,\;\;\;\text{when}\;i\neq j,$$ (37)

$$R_{1,i}=a_{4,r}\left(\xi_{i}\right),\;\;R_{2,i}=b_{4,r}\left(\xi_{i}\right),\;\;R_{3,i}=c_{4,r}\left(\xi_{i}\right),$$ (38)

where **I** is an (*N*_*x*_ + 1) × (*N*_*x*_ + 1) identity matrix. The approximate solutions for *f*(*η*, *ξ*), *g*(*η*, *ξ*) and *θ*(*η*, *ξ*) are obtained by iteratively solving the matrix Eqs (29), (30) and (31), in turn, for *r* = 0, 1, 2, ….

## Results and Discussion

In this section we present numerical results of the governing system of partial differential equations, Eqs (7)–(9) obtained using the bivariate spectral relaxation method. The set of results display convergence rates and accuracy of the iterative scheme together with the effect of the different flow parameters on the skin friction coefficients and the heat transfer rate. The number of collocation points used was 60 and 15 in space (*η*) and time (*ξ*) respectively, if not stated. The choice of these collocation points gave consistent results which did not change to a significant level when the values were increased. To monitor the convergence of the method we define the following solution error

$$\begin{array}{c} E_{f}=\max_{0\leq i\leq N_{\xi }}{||F_{r+1,i}-F_{r,i}||}_{\infty },\;\;E_{g}=\max_{0\leq i\leq N_{\xi }}{||G_{r+1,i}-G_{r,i}||}_{\infty },\\ E_{\theta }=\max_{0\leq i\leq N_{\xi }}{||\Theta_{r+1,i}-\Theta_{r,i}||}_{\infty }. \end{array}$$ (39)

The errors defined by Eq (39) can be considered to be solution based errors and they measure the number of correct digits in the approximate solutions at the *r*-th iteration level. If the numerical scheme is converging, the norms given in Eq (39) are expected to decrease with an increase in the number of iterations.

**Fig 1:**
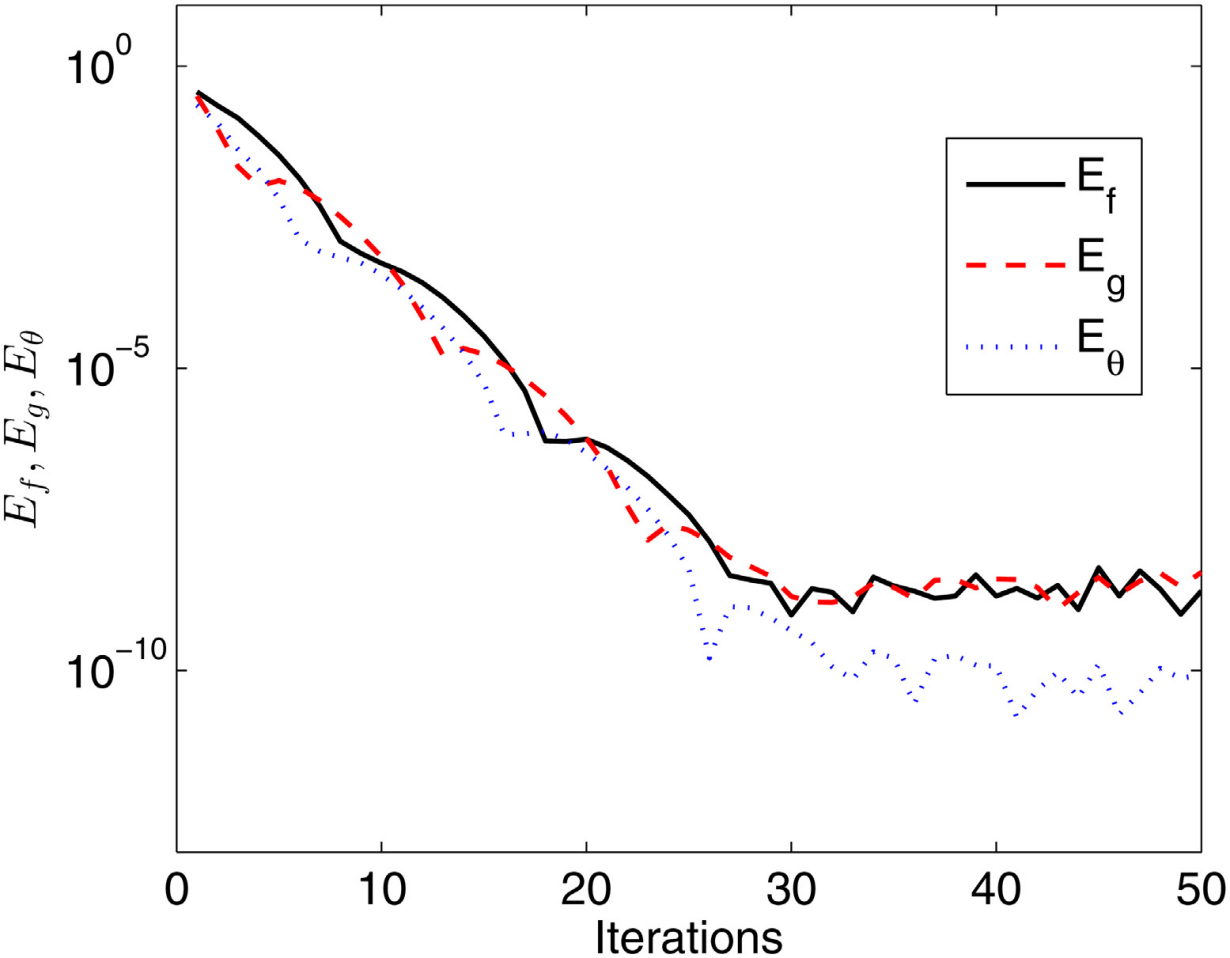
Variation of solution errors against iterations when *β* = 0.1, *β*_1_ = 0.1, *β*_2_ = 0.1, *ϵ* = 0.1, *Pr* = 0.8, *S* = 0.3.

Fig 1 presents the variation of the solution error of the approximate numerical solutions of *f*(*ξ*, *η*), *g*(*ξ*, *η*), and *θ*(*ξ*, *η*) against iterations. The monotonic decrease in all the solution errors indicates that the method converges. Full convergence is said to have been reached at the point when the convergence plots begin to plateau off. It can be seen from Fig 1 that full convergence is achieved after about thirty iterations for all solutions with a solution error close to 10^−10^.

**Fig 2:**
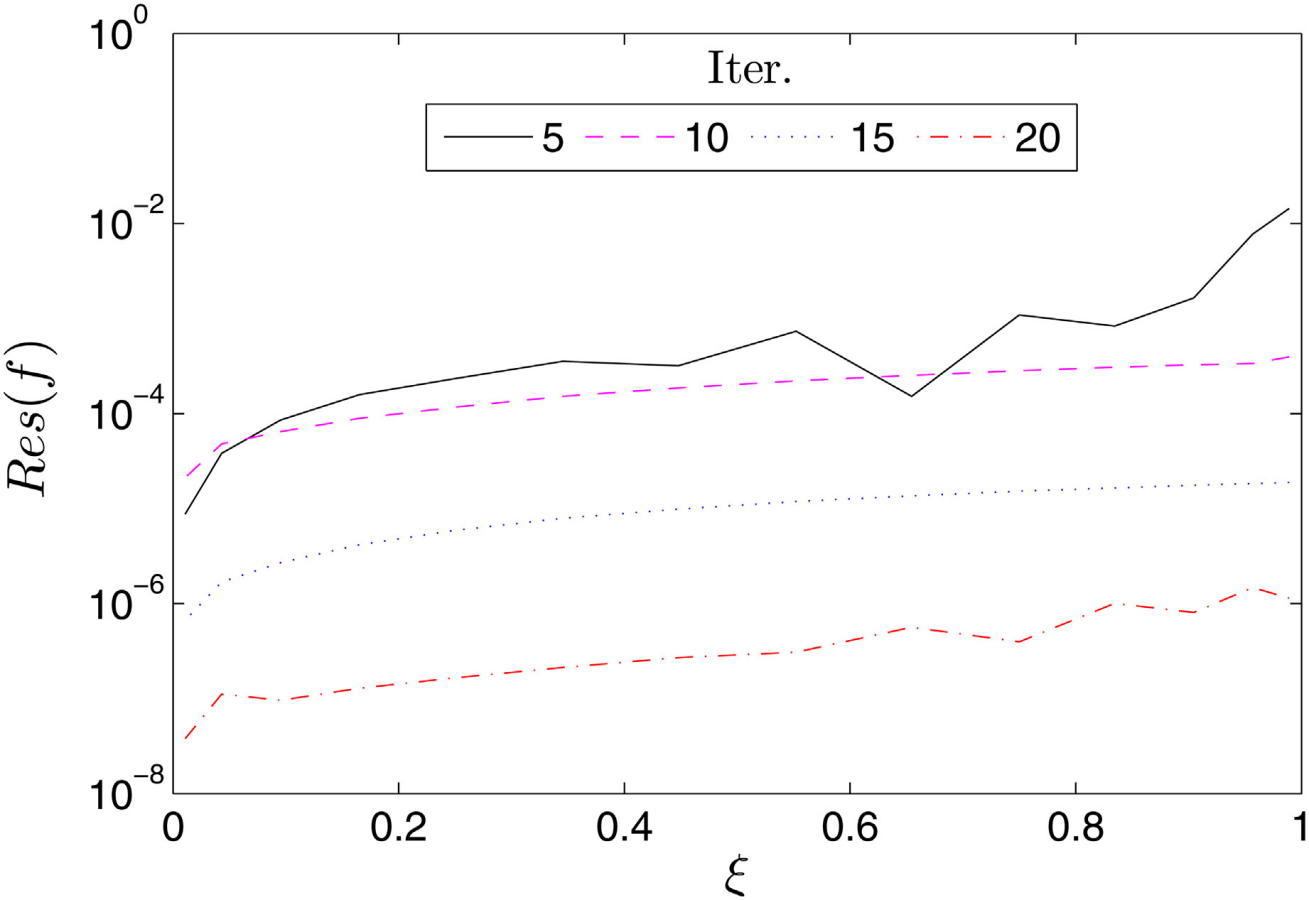
Variation of the residual error, *Res*(*f*) with *ξ*. *β* = 1, *β*_1_ = 0.1, *β*_2_ = 0.1, *ϵ* = 0.1, *Pr* = 0.8, *S* = 0.3.

**Fig 3:**
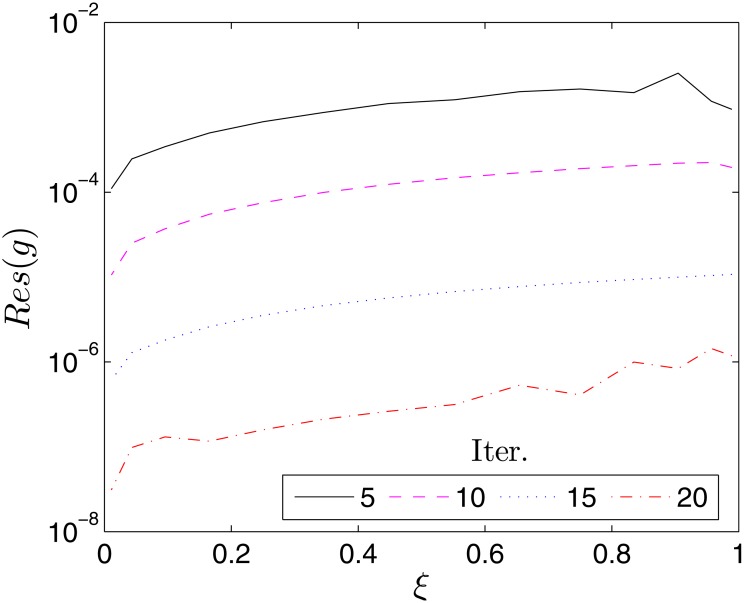
Variation of the residual error, *Res*(*g*) with *ξ*. *β* = 1, *β*_1_ = 0.1, *β*_2_ = 0.1, *ϵ* = 0.1, *Pr* = 0.8, *S* = 0.3.

**Fig 4:**
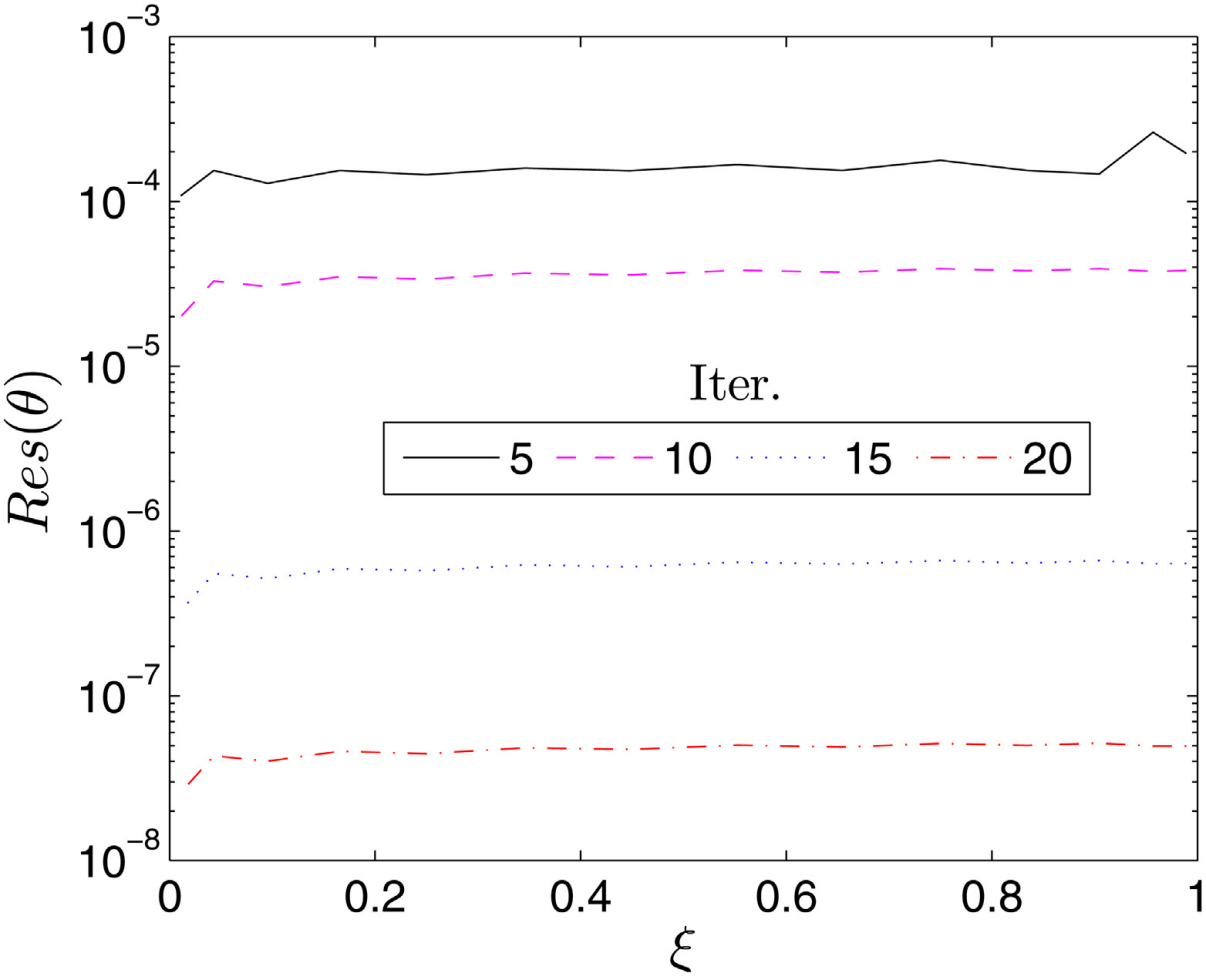
Variation of the residual error, *Res*(*θ*) with *ξ*. *β* = 1, *β*_1_ = 0.1, *β*_2_ = 0.1, *ϵ* = 0.1, *Pr* = 0.8, *S* = 0.3.

The accuracy of the proposed BSRM can be estimated by considering the residual error which measures the extent to which the numerical solutions approximate the true solution of the governing differential Eqs (7)–(9). Accordingly, we define the following residual error functions

$$\begin{array}{c} Res\left(f\right)={||\Delta_{f}\left[F_{i},G_{i},\Theta_{i}\right]||}_{\infty },\;\;Res\left(g\right)=\Delta_{g}{[F_{i},G_{i},\Theta_{i}]||}_{\infty }\\ Res\left(\theta \right)=\Delta_{f}{[F_{i},G_{i},\Theta_{i}]||}_{\infty } \end{array}$$ (40)

where Δ_*f*_, Δ_*g*_ and Δ_*f*_ represent the governing nonlinear PDEs Eqs (7), (8) and (9), respectively, and **F**_*i*_, **G**_*i*_, **Θ**_*i*_ are the BSRM approximate solutions at the time collocation points *ξ*_*i*_. Figs 2–4 show the variation of the residual errors over the time scale *ξ* for different number of iterations. The decrease in the residual errors with an increase in the number of iterations is an indication of the convergence of the method. It can also be noted that the residual error is nearly uniform across *ξ*. This is an advantage of the proposed method over some other methods whose accuracy deteriorates when *ξ* becomes large.

To validate the accuracy of the BSRM the present results are compared against exact results for −*f*′′(0, 1) and −*g*′′(0, 1) that were reported in Ariel [19] for various values of *β* in Table 1. We remark that the present problem reduces to that of [19] when *β*_1_ = *β*_2_ = 0 and *ξ* = 1. The results presented in Table 1 were computed using *N*_*x*_ = 45 and *N*_*t*_ = 10. It can be seen from the table that the BSRM gives very accurate results which are in good agreement with the exact solution for all values of the physical parameters. Table 1 also presents the computational time and the number of iterations it takes for the method to give results that are accurate to six decimal digits. It can be noted that the method gives accurate results in less than two seconds for relatively few iterations. These are some of the benefits of the proposed method. The number of iterations required to achieve the level of accuracy displayed in Table 1 may be higher than other traditional methods used to solve similar problems, such as Newton-Raphson based implicit finite difference schemes which are well-known to have quadratic convergence rates. However, the use of the relaxation idea to decouple the system of equations and spectral collocation (which require very few grid points than competing numerical methods) to discretize all variables ensures that the proposed method achieves the target level of accuracy rapidly.

**Table 1:** Variation of −*f*′′(0, 1) and −*g*′′(0, 1) against *β*.

*β*	iter.	BSRM -f′′(0, 1)	Exact [19] -f′′(0)	BSRM -g′′(0, 1)	Exact [19] -g′′(0)	time (s)
0.1	50	1.020260	1.020260	0.066847	0.066847	2.25
0.2	36	1.039496	1.039495	0.148737	0.148737	1.43
0.3	30	1.057955	1.057955	0.243360	0.243360	1.24
0.4	25	1.075788	1.075788	0.349209	0.349209	1.19
0.5	22	1.093095	1.093095	0.465205	0.465205	0.83
0.6	18	1.109947	1.109947	0.590529	0.590529	0.75
0.7	20	1.126397	1.126398	0.724532	0.724532	0.75
0.8	20	1.142488	1.142489	0.866683	0.866683	0.76
0.9	20	1.158254	1.158254	1.016539	1.016539	0.75
1.0	20	1.173720	1.173721	1.173721	1.173721	0.78

**Fig 5:**
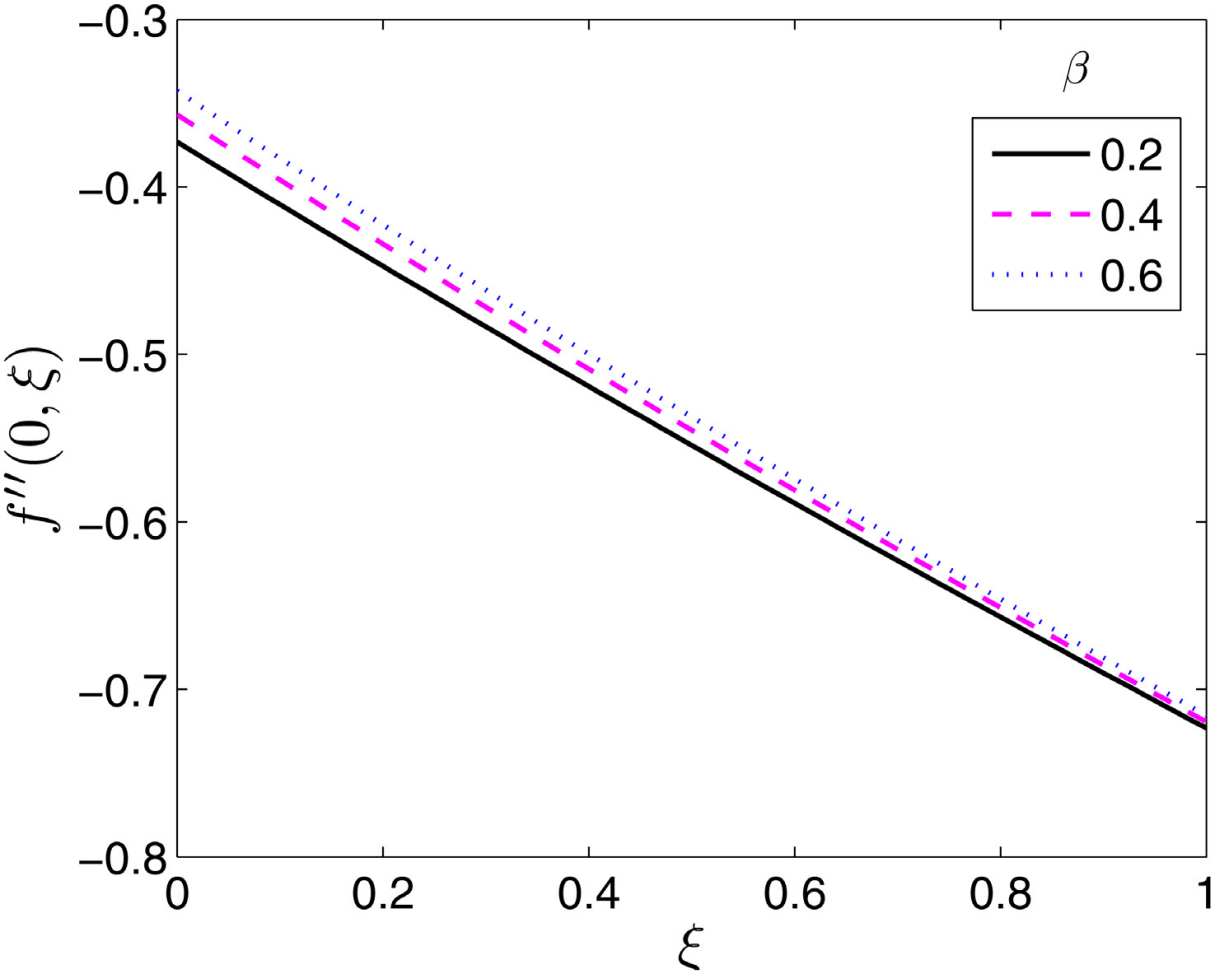
Effect of *β* on *f*′′(0, *ξ*). *β*_1_ = 0.1, *β*_2_ = 1, *ϵ* = 0.4, *Pr* = 2, *S* = 0.2.

**Fig 6:**
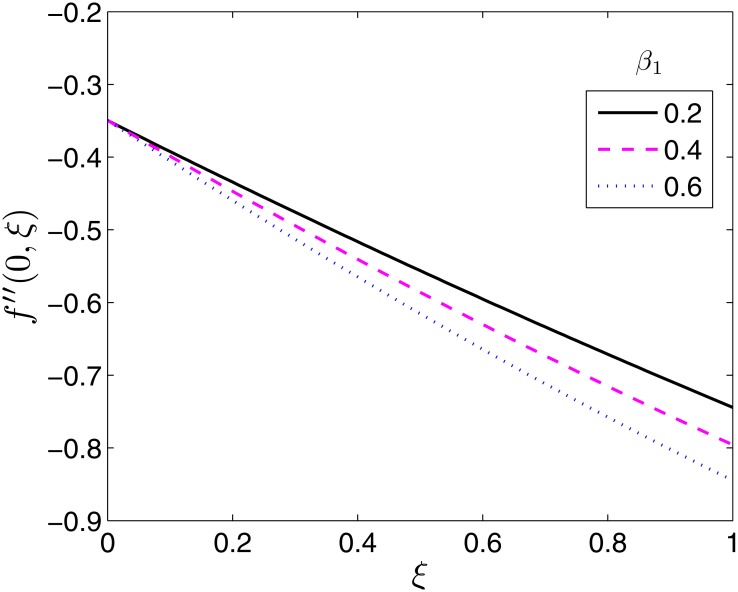
Effect of *β*_1_ on *f*′′(0, *ξ*). *β* = 0.5, *β*_2_ = 1, *ϵ* = 0.4, *Pr* = 2, *S* = 0.2.

**Fig 7:**
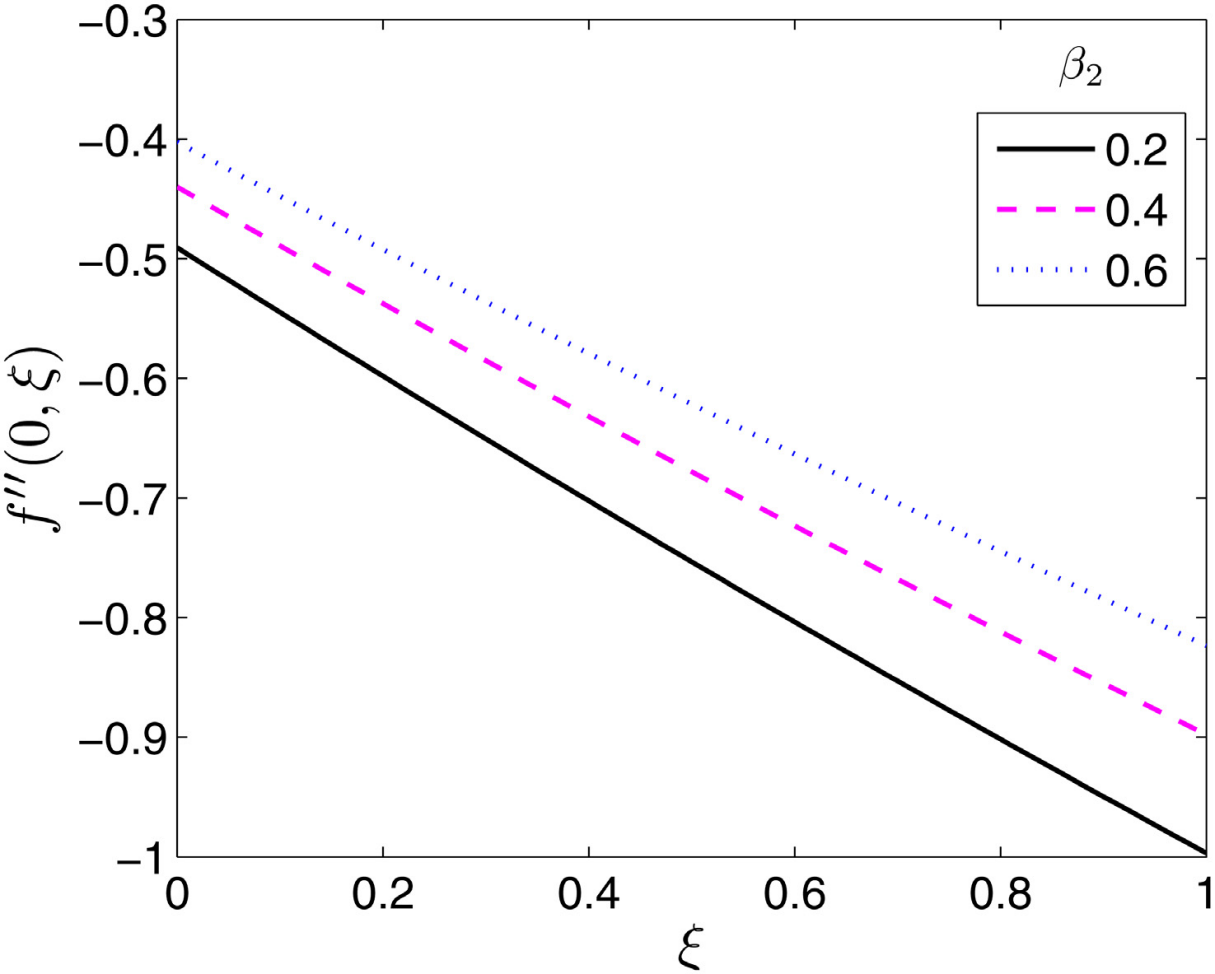
Effect of *β*_2_ on *f*′′(0, *ξ*). *β* = 0.5, *β*_1_ = 0.1 *ϵ* = 0.4, *Pr* = 2, *S* = 0.2.

The effect of the flow parameters on the velocity and temperature profiles are not presented in this study since at any fixed time they are qualitatively similar to those presented in the study done by Shehzad et al. [14] for the equivalent steady state flow case. Hence we show the effect of the flow parameters on the skin friction coefficients and Nusselt number for varying time values. The effects of the ratio parameter *β* and the Deborah numbers *β*_1_ and *β*_2_ on the local skin friction coefficient along *x*−, −*f*′′(0, *ξ*) are presented in Figs 5–7. The skin friction coefficient along *x*− is seen to be a decreasing function of time as seen across all Figs 5–7.

Fig 5 presents the behaviour of −*f*′′(0, *ξ*) with the ratio of stretching rates parameter *β*. Physically, increasing *β* implies an increase in the velocity field along *y*−. This explains why the local skin friction along *x*− is not significantly affected by changes in *β*. The effect is almost negligible towards the steady state.

The effect of the Deborah number *β*_1_ is to increase the local skin friction coefficient as seen in Fig 6. The effect is significantly pronounced towards *ξ* = 1. Physically, *β*_1_ is dependant on the relaxation time of the Oldroyd B fluid. Increasing *β*_1_ directly increases the relaxation time which in turn reduces the velocity of the fluid near the sheet surface. This results in an increase in the thickness of the hydrodynamic boundary layer which causes the local skin friction coefficient to increase. On the other hand, *β*_2_ is directly dependent on the retardation time of the fluid. Hence with increase in *β*_2_, the local skin friction coefficient decreases because of the reduced hydrodynamic boundary layer caused by an increase in the velocity of the fluid near the surface of the sheet. This effect is shown in Fig 7.

**Fig 8:**
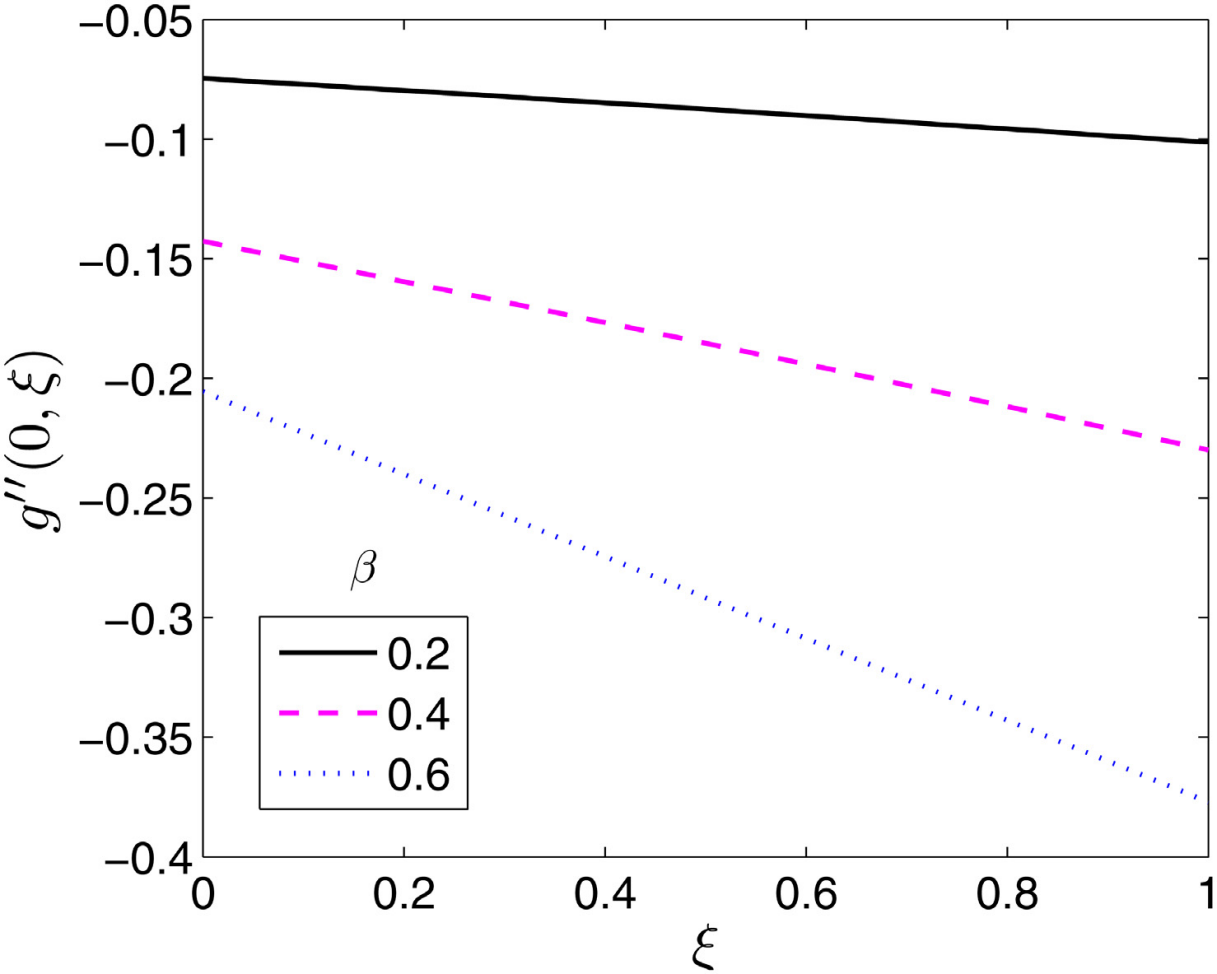
Effect of *β* on *g*′′(0, *ξ*). *β*_1_ = 0.1, *β*_2_ = 1, *ϵ* = 0.4, *Pr* = 2, *S* = 0.2.

**Fig 9:**
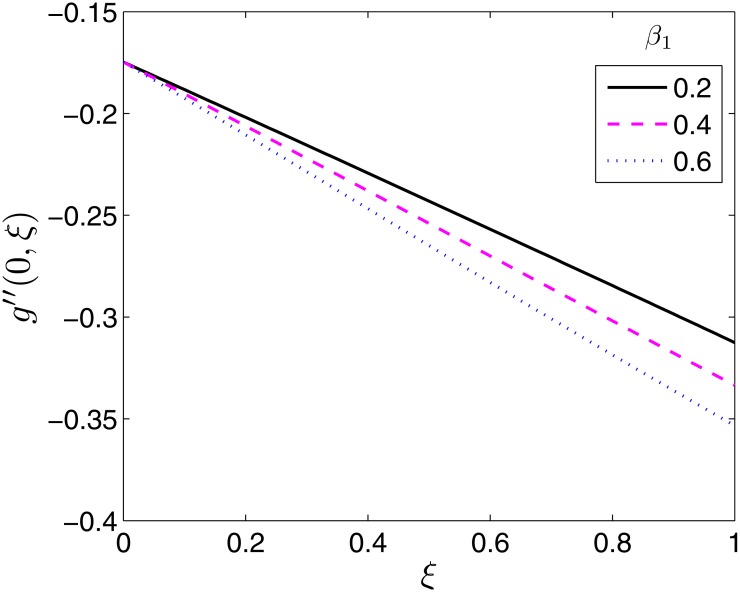
Effect of *β*_1_ on *g*′′(0, *ξ*). *β* = 0.5, *β*_2_ = 1, *ϵ* = 0.4, *Pr* = 2, *S* = 0.2.

**Fig 10:**
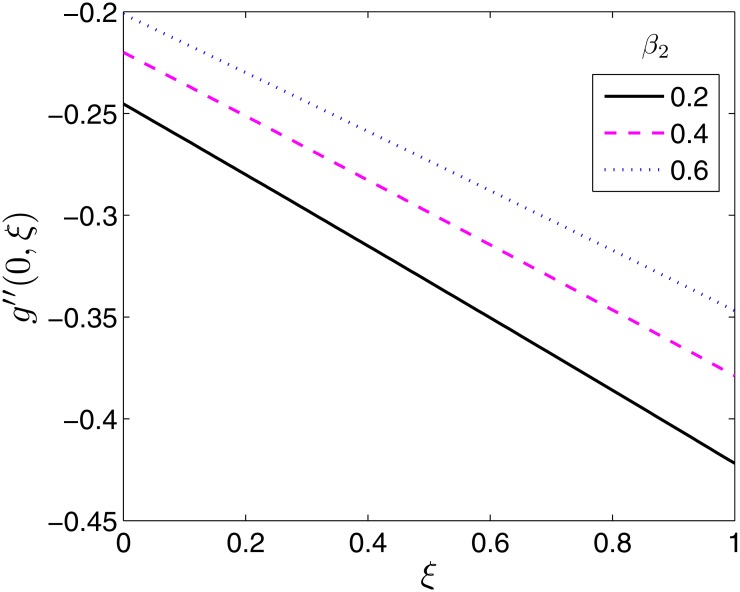
Effect of *β*_2_ on *g*′′(0, *ξ*). *β* = 0.5, *β*_1_ = 0.1 *ϵ* = 0.4, *Pr* = 2, *S* = 0.2.

Figs 8–10 demonstrate the behaviour of the local skin friction coefficient along the *y*− direction, −*g*′′(0, *ξ*) as a function of time for varying values of *β*, *β*_1_ and *β*_2_ respectively.

As expected, the effect of *β* is to increase −*g*′′(0, *ξ*) as shown in Fig 8. On the contrary to −*f*′′(0, *ξ*) the effect is significantly pronounced at all times. The effect of the Deborah numbers on −*g*′′(0, *ξ*) is qualitatively similar to those they have on −*f*′′(0, *ξ*) as shown in Figs 9 and 10. The effect of *β*_1_ is to increase −*g*′′(0, *ξ*) while that of *β*_2_ is to decrease −*g*′′(0, *ξ*). The physical implication is similar to that of −*f*′′(0, *ξ*) in Figs 6 and 7.

**Fig 11:**
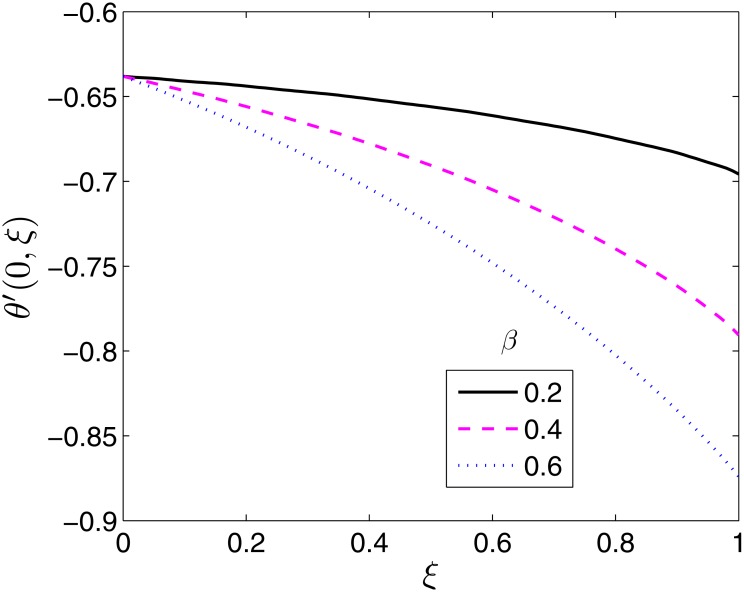
Effect of *β* on *θ*′(0, *ξ*). *β*_1_ = 0.1, *β*_2_ = 1, *ϵ* = 0.4, *Pr* = 2, *S* = 0.2.

**Fig 12:**
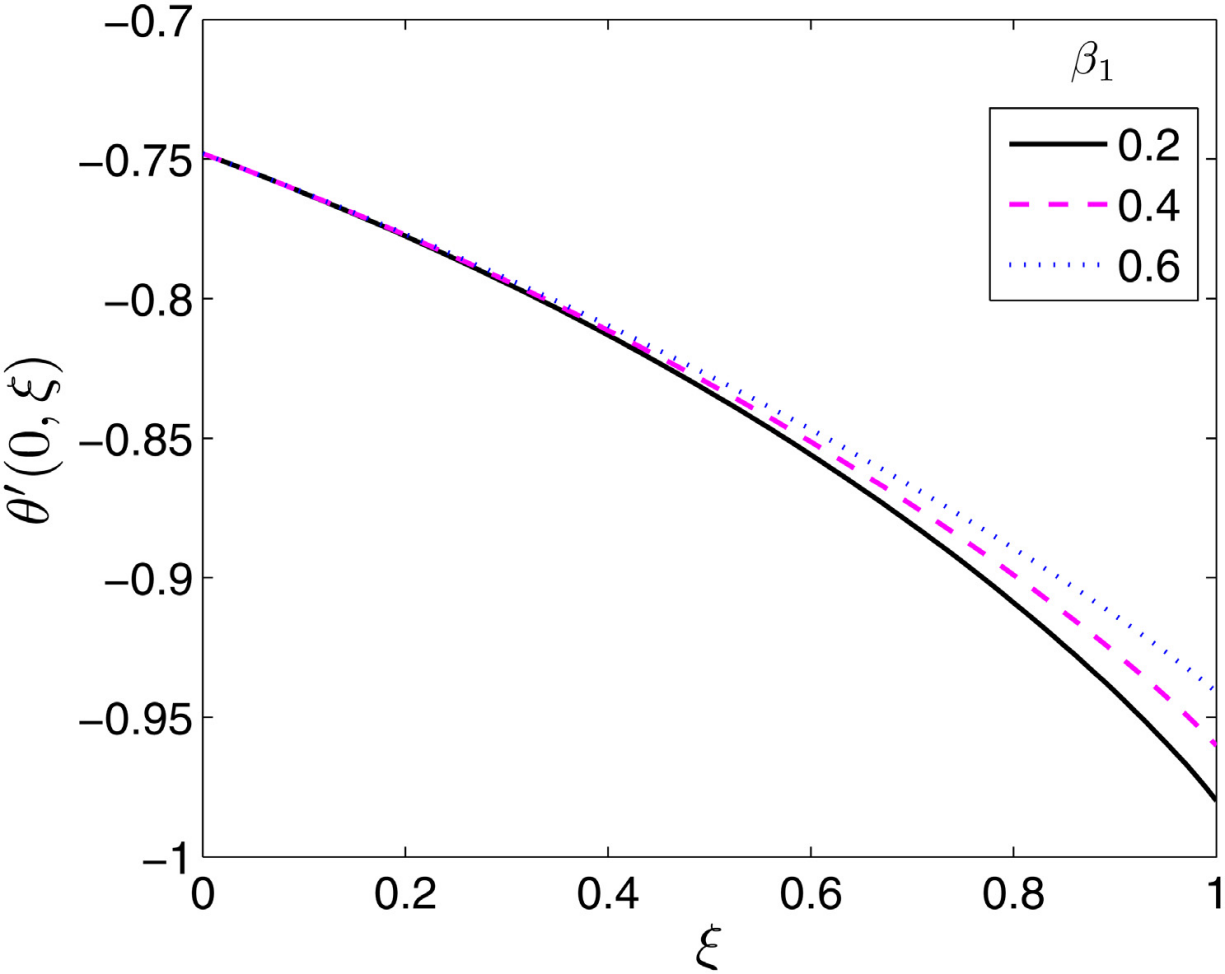
Effect of *β*_1_ on *θ*′(0, *ξ*). *β* = 0.5, *β*_2_ = 1*ϵ* = 0.4, *Pr* = 2, *S* = 0.2.

**Fig 13:**
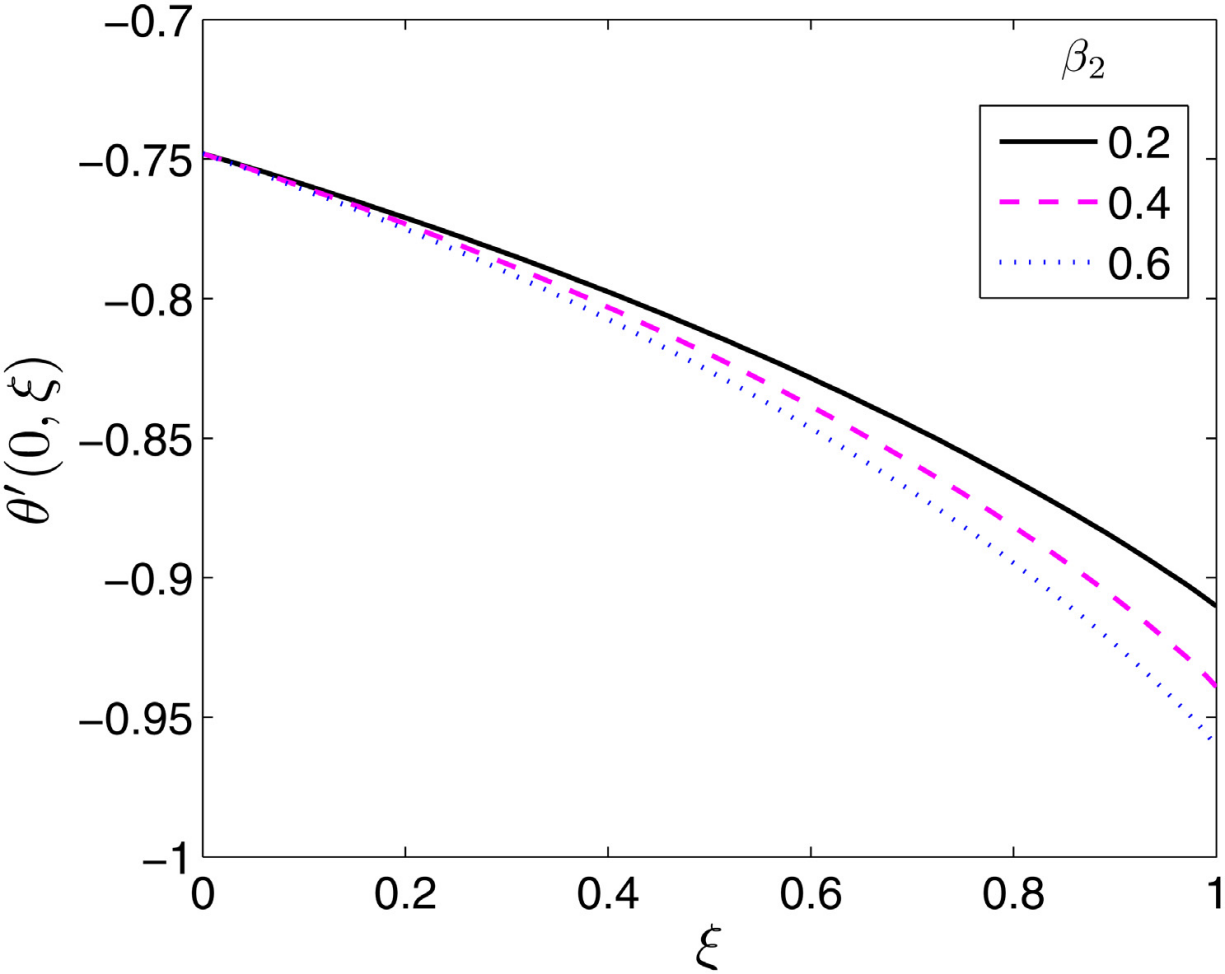
Effect of *β*_2_ on *θ*′(0, *ξ*). *β* = 0.5, *β*_1_ = 0.1, *ϵ* = 0.4, *Pr* = 2, *S* = 0.2.

**Fig 14:**
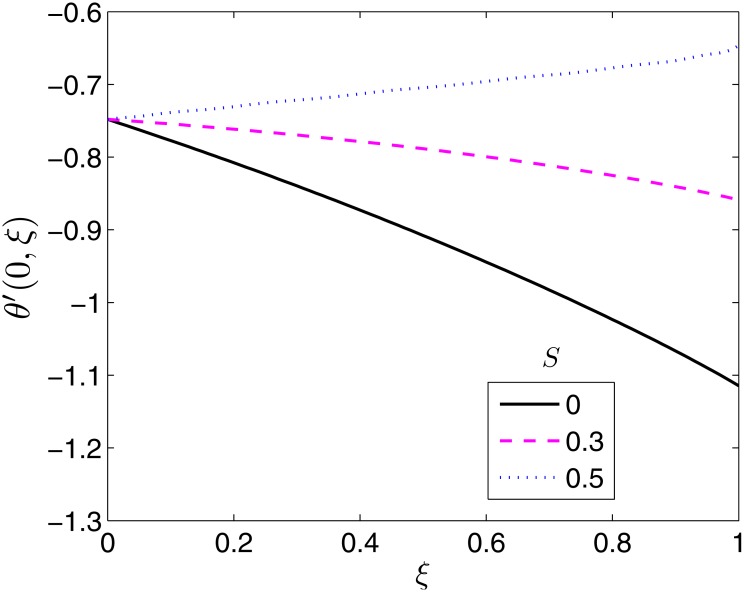
Effect of *S* on *θ*′(0, *ξ*). *β* = 0.5, *β*_1_ = 0.5, *β*_2_ = 1*ϵ* = 0.1, *Pr* = 2.

**Fig 15:**
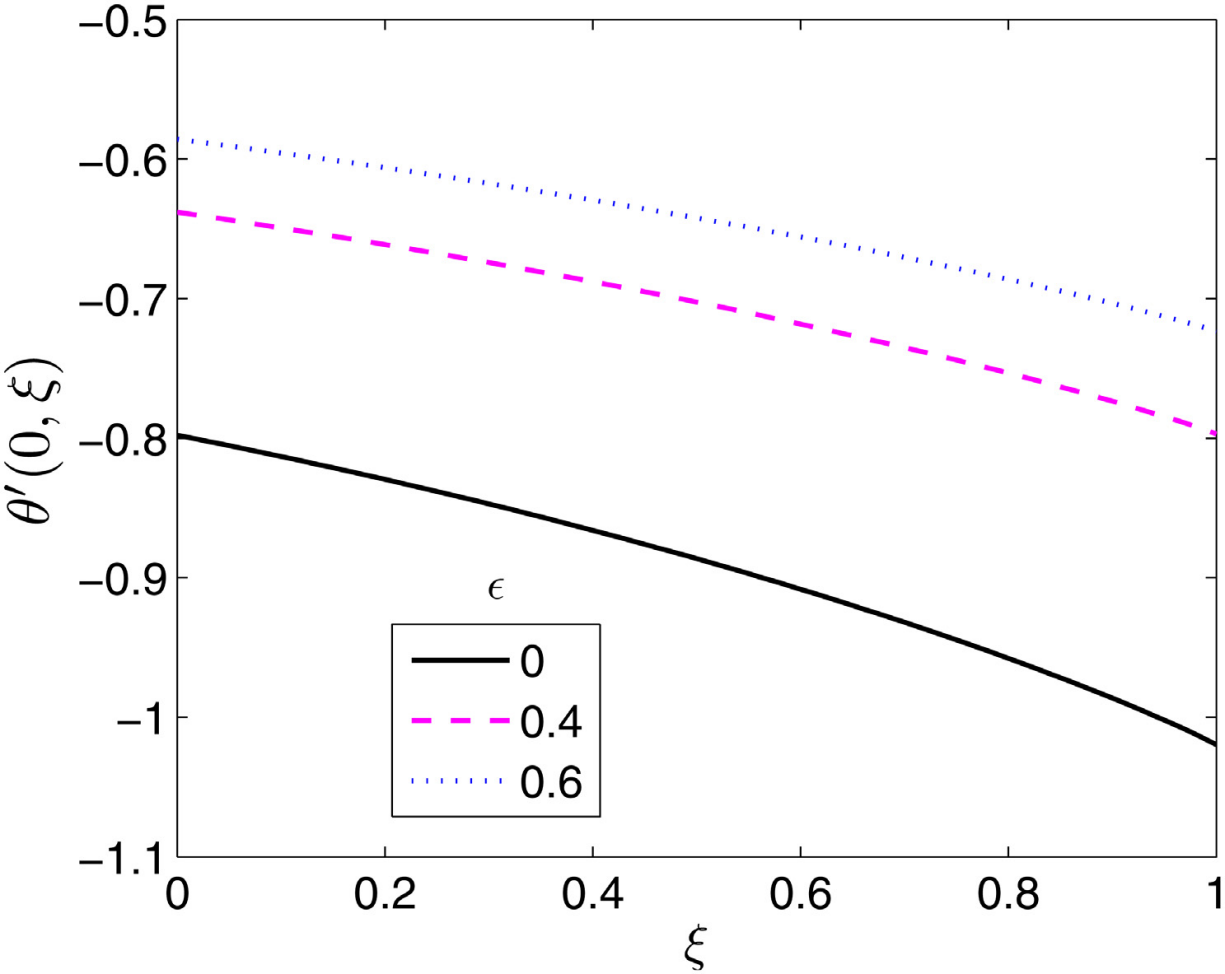
Effect of *Pr* on *θ*′(0, *ξ*). *β* = 0.5, *β*_1_ = 0.5, *β*_2_ = 1*ϵ* = 0.1, *S* = 0.2.

**Fig 16:**
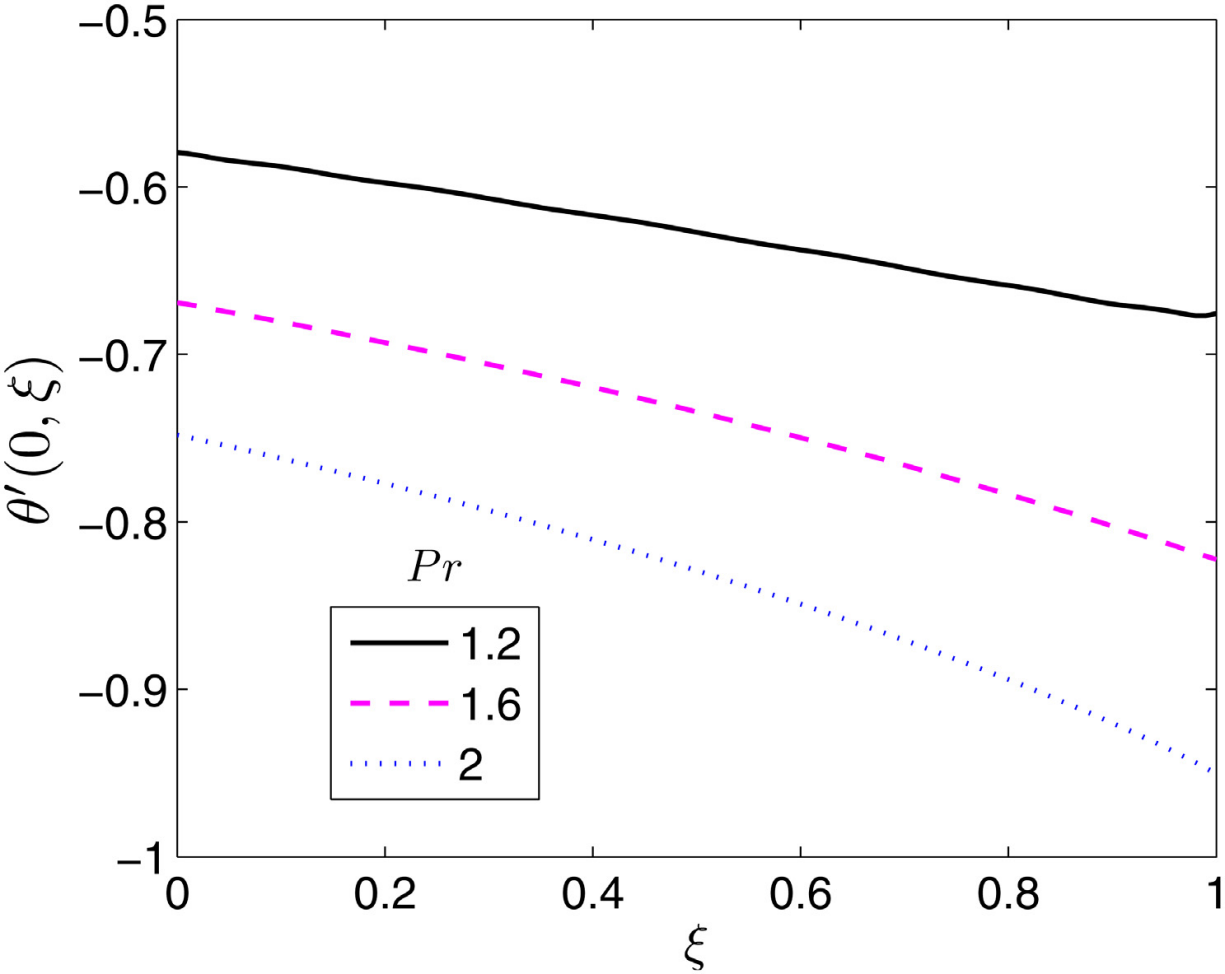
Effect of *ϵ* on *θ*′(0, *ξ*). *β* = 0.5, *β*_1_ = 0.5, *β*_2_ = 1 *S* = 0.2, *Pr* = 2.

The effects of the different flow parameters on the surface heat transfer rate, −*θ*′(0, *ξ*) are displayed in Figs 11–16. In all the figures, the Nusselt number is shown to be an increasing function of time (moving from *ξ* = 0 to *ξ* = 1).

Variation of the Nusselt number with the ratio of stretching rates parameter, *β* is shown in Fig 11. The heat transfer rate is seen to increase with increase in *β*. The temperature field and thermal boundary layer decrease with in *β* resulting in increased heat transfer rates.

Fig 12 presents the effect of *β*_1_ on the Nusselt number. Increasing the relaxation time leads to increase in the temperature field and boundary layer thickness. The rate of heat transfer is thus reduced as shown in Fig 12. Retardation has the opposite effect on the temperature and thermal boundary layer thickness. Retardation tends to provide resistance which causes a reduction in both the temperature field and the thermal boundary layer. This explains why the Nusselt number increases with increase in *β*_2_ as seen in Fig 13.

Fig 14 presents the effect of heat generation on the surface heat transfer rate as a function of time. At the start of the motion, the heat transfer rate is independent of heat generation. The heat transfer rate is seen to be high when there is no enhancement (*S* = 0) to the temperature field of the fluid. When *S* = 0, the temperature differences between the two mediums are large. Heat transfer rates decrease with increase in *S* because the heat generation mechanism increases the temperature of the fluid near the surface of the sheet.

The effect *ϵ* on the Nusselt number is seen to be similar to that of *S* because both parameters have the same effect on the temperature field and thermal boundary layer. Increasing *ϵ* boosts the temperature and thermal boundary layer thickness, in turn decreasing the heat transfer rate as shown in Fig 15. The difference is that contrary to *S*, the Nusselt number is dependent on *ϵ* across all values of *ξ*. This occurs because the temperature varies slowly and decays rapidly for *ϵ* in comparison to *S* [14].

The effect of the Prandtl number on the surface heat transfer rate is shown in Fig 16. The heat transfer rate is seen to increase with increase in *Pr*. Physically, this is caused by the fact that increasing *Pr* reduces the temperature field and the thermal boundary layer thickness, in turn increasing the heat transfer rate. The increase with *ξ* is almost linearly and significantly pronounced for values of *ξ*.

**Fig 17:**
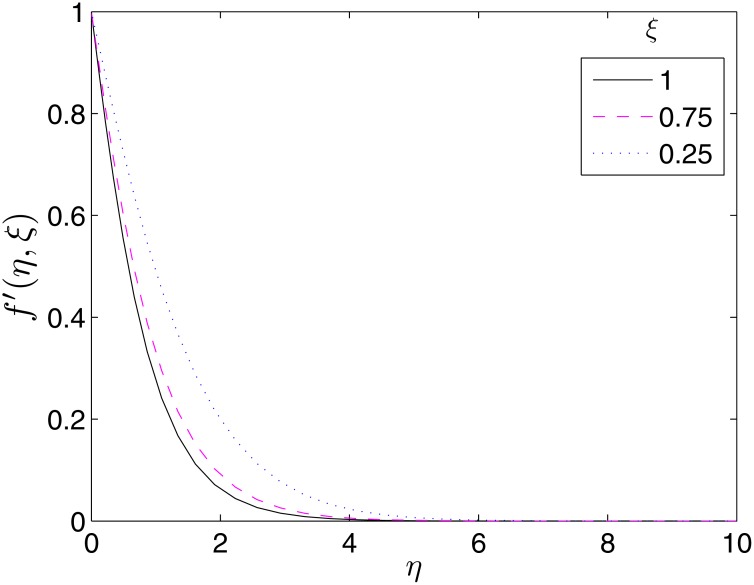
Variation of *f*′(*η*, *ξ*) with *ξ*. *β* = 1, *β*_1_ = 0.5, *β*_2_ = 0.5, *ϵ* = 1, *Pr* = 2, *S* = 0.1.

**Fig 18:**
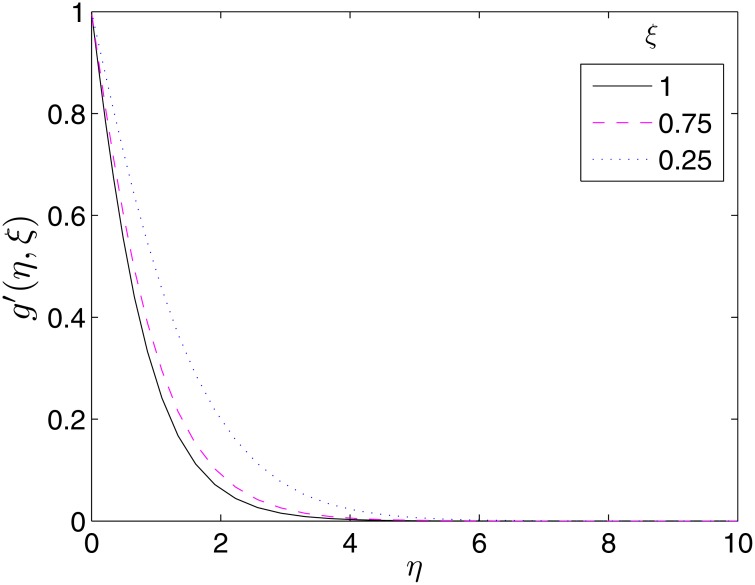
Variation of *g*′(*η*, *ξ*) with *ξ*. *β* = 1, *β*_1_ = 0.5, *β*_2_ = 0.5, *ϵ* = 1, *Pr* = 2, *S* = 0.1.

**Fig 19:**
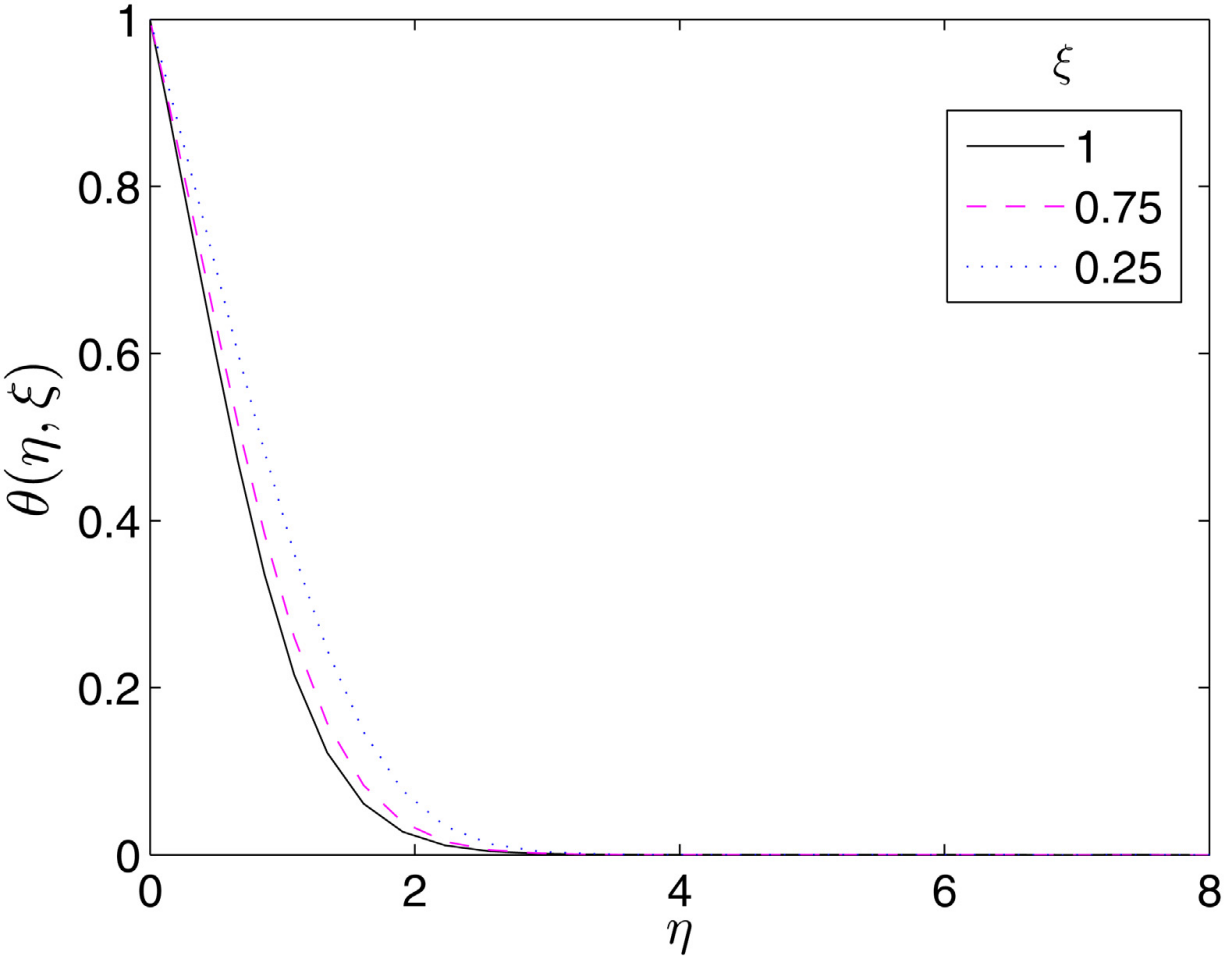
Variation of *θ*(*η*, *ξ*) with *ξ*. *β* = 1, *β*_1_ = 0.5, *β*_2_ = 0.5, *ϵ* = 1, *Pr* = 2, *S* = 0.1.

To validate the importance of the study, we present Figs 17–19 where the velocity and temperature profiles are shown at different time levels. It is important to have an idea what happens to the velocity and temperature of the fluid overtime for proper precautions to be taken in experiments. From the Figures, it can be seen that these flow properties are decreasing functions of time.

## Conclusions

The present study extended the application of a bivariate spectral relaxation method to systems of nonlinear partial differential equations. For the numerical experimentation, a highly nonlinear coupled system of PDEs governing the unsteady flow of a three-dimensional Oldroyd-B fluid with variable thermal conductivity and heat generation was solved. Numerical simulations were carried out successfully, showing convergence behaviour and accuracy of the iterative scheme. In addition, numerical simulations were carried out showing the effect of the different flow parameters on the skin friction coefficients and the heat transfer rate. Unsteadiness was found to have a significant influence on all flow properties. The observations made were found to be consistent with other results in similar studies reported in the literature. The method proved great efficiency in solving the system making it a potential tool for numerical solutions of nonlinear systems of PDEs. The success of the study provokes the need of further numerical experiments on the method involving different types of partial differential equations.
